# Supplementary material for: Region-specific mechanosensation modulates Drosophila postural control behaviour
Source: eLife. 2026 Sep 23;14:RP108505. doi: 10.7554/eLife.108505 (PMC13600696; doi:10.7554/eLife.108505)
Supplement: Peer review history

# Editor report: eLife Assessment

**DOI:** 10.7554/eLife.108505.3.sa0

**Authors:** Sonia Q Sen

**Affiliations:** Tata Institute for Genetics and Society India

**Keywords:** Compelling, Important

This study by Roseby and colleagues shows that region-specific mechanosensation - especially anterior-dorsal inputs - controls larval self-righting, and links this to Hox gene function in sensory neurons. The work is **important** for understanding how body plan cues shape sensorimotor behaviour, and the experimental toolkit will be of use to others. The strength of evidence is **compelling** with respect to the assays developed and the involvement of the anterior region, the evidence is more limited with respect to the dorso-ventral organization of sensory inputs in that region and the mechanism by which Hox genes contribute to the process. These findings will be of broad interest to researchers studying neural circuits, developmental genetics, and the evolution of behaviour.

# Referee report: Reviewer #1 (Public review)

**DOI:** 10.7554/eLife.108505.3.sa1

Summary:

Roseby and colleagues report on a body region-specific sensory control of the fly larval righting response, a body contortion performed by fly larvae to correct their posture from an inverted (dorsal side down) position. This is an important topic because of the general need for animals to locomote in the correct orientation and the clever and broadly useful methodologies used in this paper to uncover the sensory triggers for the behavior, including a body region-specific optogenetic approach along different axial positions of the larva, region-specific manipulation of surface contacts with the substrate, and a 'water unlocking' technique to initiate righting behaviors, all strengths of the manuscript. The authors found that multidendritic neurons, particularly the daIV neurons, are necessary for righting behavior. The contribution of daIV neurons had been shown by the authors in a prior paper (Klann et al, 2021), but that study had used constitutive neuronal silencing. Here the authors used acute inactivation to confirm this finding. Additionally, the authors describe an important role for anterior sensory neurons. They move on to test the genetic basis for righting behavior and, consistent with the regional specificity they observe, implicate sensory neuron expression of Hox genes Antennapedia and Abdominal-b in self-righting.

Strengths:

Strengths of this paper include the important question addressed and the elegant and innovative combination of methods, which led to clear insights into the sensory biology of self-righting and links between body plan and nervous system function that will be useful for others in the field. The manuscript is very clearly written and couched in interesting biology.

Limitations:

There are several important questions for future study that, left unresolved, do not diminish the significance of this manuscript. These include the cellular and developmental basis for Hox gene action, the contributions of dorsal and ventral regions of the animal in righting, and the regional contributions of other sensory cell types in the righting response.

Comments on revised version.

The authors have addressed my major concerns.

# Referee report: Reviewer #2 (Public review)

**DOI:** 10.7554/eLife.108505.3.sa2

Summary

This work explores the relationship between body structure and behavior by studying self-righting in Drosophila larvae, a conserved behavior that restores proper orientation when turned upside-down. The authors first introduce a novel "water unlocking" approach to induce self-righting behavior in a controlled manner. Then, they develop a method for region-specific inhibition of sensory neurons revealing that anterior, but not posterior, sensory neurons are essential for proper self-righting. Deep-learning-based behavioral analysis shows that anterior inhibition prolongs self-righting by shifting head movement patterns, indicating a behavioral switch rather than a mere delay. Additional genetic and molecular experiments demonstrate that specific Hox genes are necessary in sensory neurons, underscoring how developmental patterning genes shape region-specific sensory mechanisms that enable adaptive motor behaviors.

Strengths

The work by Roseby et al. is notable for its elegant experimental design, the development of innovative methods that are likely to benefit the fly behavior community, and the strong experimental support for its conclusions. The manuscript is clearly written, well structured, and presents thoughtfully designed experiments that have been further improved in the revised version. This updated manuscript includes a comprehensive set of behavioral experiments using an additional Gal4 line (ppk-Gal4), which yields confirmatory results and strengthens support for the original hypothesis. It also incorporates quantification of Gal4 line strength, improvements to existing figures, the addition of new figures, and overall refinement of the text.

Weakness:

A remaining limitation of this manuscript is the lack of a cellular and mechanistic analysis explaining how Hox genes give rise to the observed behavioral phenotypes. The authors note that this question is being addressed in an ongoing follow-up study, which will expand the project to examine the roles of all Hox genes across the sensory system and to characterize their expression patterns within each of its subcomponents, with the aim of providing mechanistic insight. I look forward to seeing this work in a future manuscript.

Comments on revised version.

I have no further recommendations for the authors; most of my comments and questions have been satisfactorily addressed.

# Author comment: Author response

**DOI:** 10.7554/eLife.108505.3.sa3

**Authors:** William Roseby, Jonathan AC Menzies, Victoria A Lipscomb, Claudio R Alonso

**Affiliations:** Imperial College London London United Kingdom; University of Sussex Brighton United Kingdom; University of Sussex Brighton United Kingdom; University of Sussex Brighton United Kingdom

The following is the authors’ response to the original reviews.

**Public Reviews:**

**Reviewer #1 (Public review):**

Strengths:

Strengths of this paper include the important question addressed and the elegant and innovative combination of methods, which led to clear insights into the sensory biology of self-righting, and that will be useful for others in the field. This is a substantial contribution to understanding how animals correct their body position. The manuscript is very clearly written and couched in interesting biology.

Limitations:

(1.1) The interpretation of functional experiments is complicated by the proposed excitatory and inhibitory roles of dorsal and ventral sensory neuron activity, respectively. So, while silencing of an excitatory (dorsal) element might slow righting, silencing of inputs that inhibit righting could speed the behavior. Silencing them together, as is done here, could nullify or mask important D-V-specific roles. Selective manipulation of cells along the D-V axis could help address this caveat.

We highly appreciate the thoughtful comments by Rev1 pointing out the relative simplicity of our current inferences regarding the role of dorsal vs. ventral substrate contact, and agree with the suggestion that cells along the DV axis could have diverse roles in their contribution to self-righting. In this context, we wish to point out two aspects, one theoretical and one practical. Regarding theory, our view is that this may not be a simple case of “excitation vs. inhibition”, but rather one in which the coordinated and dynamic activity of distributed sensory neurons promotes differential action selection in alignment with environmental conditions – a framework that could involve many different behaviours with a still uncertain level of granularity (e.g., is self-righting different if the larva is rotated to 160º instead of exactly 180º?). Regarding the practical aspect, while this area represents a fascinating point for future investigation, it is currently limited by technological development, particularly in the context of this study where a relatively low-cost implementation has been used to probe the AP axis. Investigation of the DV axis would require further technological development, since optogenetic light would need to be precisely delivered from the side rather than from underneath, with a greater degree of resolution compared to the AP axis given the much smaller width of the larva (~120-140µm) relative to its length (~550-600µm). Therefore, whilst we appreciate these comments and suggestion, we believe this line of experiments is ideal for a follow-up investigation, rather than being implemented in the current study.

(1.2) Prior studies from the authors implicated daIV neurons in the righting response. One of the main advances of the current manuscript is the clever demonstration of region-specific roles of sensory input. However, this is only confirmed with a general md driver, 190(2)80, and not with the subsetspecific Gal4, so it is not clear if daIV sensory neurons are also acting in a regionally-specific manner along the A-P axis.

To address this interesting and important comment by Rev1 we have carried out a new experiment using an alternative driver to *109(2)80-Gal4* and testing the impact of these manipulations on larval behaviour. The revised version of our MS includes a new figure Supp Fig S3 which shows self-righting times when using the *ppk-Gal4* driver with the opto-axial technique. As observed with the *109(2)80-Gal4* driver, self-righting was delayed in anterior but not posterior inhibition conditions, suggesting the daIV neurons act in a region-specific manner to trigger postural control behaviour.

We have also conducted a head casting analysis in the ppk domain; in another new figure, Supp Fig S7, we also show that head casting behaviour is also increased in the same manner as with the *109(2)80-Gal4* driver.

These new panels and figures are cited within the sub sections entitled “Optogenetic inhibition of anterior but not posterior multidendritic neurons delays self-righting” and “Inhibition of anterior multidendritic neurons is associated with increased head casting during self-righting”, on pages 25 and 28, respectively. We are grateful to Rev1 for this suggestion, which we consider qualitatively improves our paper.

(1.3) The manuscript is narrowly focused on sensory neurons that initiate righting, which limits the advance given the known roles for daIV neurons in righting. With the suite of innovative new tools, there is a missed opportunity to gain a more general understanding of how sensory neurons contribute to the righting response, including promoting and inhibiting righting in different regions of the larva, as well as aspects of proprioceptive sensing that could be necessary for righting and account for some of the observed effects of 109(2)80.

Once again, we appreciate this interesting comment by Rev1. We feel our study provides novelty in understanding how sensory neurons in different body regions contribute to the induction of the behaviour. We developed new technology to show that the activity of anterior sensory neurons is essential for normal righting and inhibiting this activity leads to a switch to a different behavioural regime. We feel this represents a substantial advancement in our understanding of how this behaviour is initiated that has not been previously described. Whilst we also appreciate there is likely to be a substantial role of proprioception in self-righting behaviour, our work here focuses on the external stimuli that elicit self-righting, as a detailed understanding of proprioception would be out of scope and require the development of further techniques to manipulate and measure larval posture. As detailed in the above comment, we feel that the more targeted investigation of daIV neurons can also shed some light on the cell-type specificity and inputs to the self-righting induction process.

(1.4) Although the authors observe an influence of Hox genes in righting, the possible mechanisms are not pursued, resulting in an unsatisfying conclusion that these genes are somehow involved in a certain region-specific behavior by their region-specific expression. Are the cells properly maintained upon knockdown? Are axon or dendrite morphologies of the cells disrupted upon knockdown?

We agree with this comment in that further investigating the effects of Hox expression on localised aspects of the sensory system poses an interesting line of investigation. Indeed, we are currently conducting a full scale analysis of Hox gene effects across the sensory field. As things stands, it is not clear how Hox gene expression could affect local sensory processes, a mechanism which could involve morphological changes, changes in neuronal excitability (e.g. due to changes in channel expression), synapse formation and/or efficiency, cell development and identity, and/or combinations of these effects, amongst other possibilities. It is clear that a complete and satisfying investigation of this mechanism for each of the Hox genes would pose a substantial amount of work so, while we acknowledge the merit of Rev1’s comment, we consider that adding a cellular-mechanistic analysis of Hox effects is out of scope for the present study and shall constitute a central matter for a followup study emerging from current projects. We think that our data on Hox expression/function as reported here should serve to open up the analysis of genetic regulation of local sensory function, an area in which we are currently working very actively.

(1.5) There could be many reasons for delays in righting behavior in the various manipulations, including ineffective sensory 'triggering', incoherent muscle contraction patterns, initiation of inappropriate behaviors that interfere with righting sequencing, and deficits in sensing body position. The authors show that delays in righting upon silencing of 109(2)80 are caused by a switch to head casting behavior. Is this also the case for silencing of daIV neurons, Hox RNAi experiments, and silencing of CO neurons? Does daIII silencing reduce head casting to lead to faster righting responses?

This is an insightful comment. In the revised version of the manuscript, we do indeed show that anterior inhibition of daIV neurons leads to the same head casting behaviour as with the 109(2)80 domain, which we interpret as an inability of the larvae to sense the underlying substrate (see page 28). We hope the new data addresses this comment, at least to an extent. While we acknowledge it would also be insightful to run this behavioural analysis for other experimental conditions, such as the daIII inhibition and Hox RNAi lines, these experiments pose a specific technical difficulty: the behavioural analysis relies on a deep neural network (DNN) which was trained solely on recordings of the opto-axial technique, meaning it does not translate well to other experimental situations. This problem is further compounded by the use of L1 larvae, which means recording resolution is insufficient to accurately define the body landmarks used in the posture tracking at a smaller scale. Therefore, the recourse for identifying behavioural changes is manual observation, which we feel is too inconsistent to address a quantitative question like this.

(1.6) 109(2)80 is expressed in a number of central neurons, so at least some of the righting phenotype with this line could be due to silenced neurons in the CNS. This should at least be acknowledged in the manuscript and controlled for, if possible, with other Gal4 lines.

We thank the reviewer for making this interesting comment. We have added a phrase to the section “Conditional inhibition of multidendritic neurons delays self-righting” (p21) which acknowledges the presence of 109(2)80 expression in the CNS (as reported by Hughes and Thomas). We agree that ideally, a variety of sensory Gal4 lines would be used to check for consistency of the effects. However, it is also important to note that 109(2)80 is one of the only available Gal4 lines with near sole md neuron expression, as other Gal4s also drive expression strongly in external sensory cells for example. Thus, re-running experiments with these other lines – which would involve a substantial investment of time and resources – would not be an ideal strategy. We feel that the new observation of (very) similar axial results using the ppk-Gal4, which does express solely in the daIV neurons, better helps to confirm the specificity of the findings to multidendritic neurons.

Other points:

(1.7) Interpretation of roles of Hox gene expression and function in righting response should consider previous data on Hox expression and function in multidendritic neurons reported by Parrish et al. Genes and Development, 2007.

We thank Rev1 for pointing out this study, which is definitively important to discuss given our results on Hox genes. To address this gap, we have added an additional paragraph in the Discussion (p37) to discuss the documented effects of Hox genes on da neuron dendritic morphology and how our results can be interpreted in light of this.

(1.8) The daIII silencing phenotype could conceivably be explained if these neurons act as the ventral inhibitors. Do the authors have evidence for or against such roles?

This is another interesting suggestion. If the daIII neurons were to fulfil this role, then in theory, their inhibition would result in self-righting behaviour under conditions of combined dorsal and ventral substrate contact. This is not an experiment we performed, so we are currently unable to confirm or rule out this possibility. However, we note from casual observation that daIII inhibition does not cause larvae to spontaneously self-right. As mentioned above, our view is not one in which the system has “dorsal/ventral stimulators/inhibitors” for a given behaviour, but that action selection proceeds according to a coordination of many (dynamic) contextual clues. Given the new results with the axial inhibition of daIV neurons (see above) it might be more parsimonious to suggest that these “tiling” neurons are primarily responsible for detecting substrate contact around the full circumference of the animal, rather than this involving different cell types according to the different sides of the body.

**Reviewer #2 (Public review):**

Strengths:

The work of Roseby et al. does what it says on the tin. The experimental design is elegant, introducing innovative methods that will likely benefit the fly behavior community, and the results are robustly supported, without overstatement.

Weaknesses:

The manuscript is clearly written, flows smoothly, and features well-designed experiments. Nevertheless, there are areas that could be improved. Below is a list of suggestions and questions that, if addressed, would strengthen this work:

(2.1) Figure 1A illustrates the sequence of self-righting behavior in a first instar larva, while the experiments in the same figure are performed on third instar larvae. It would be helpful to clarify whether the sequence of self-righting movements differs between larval stages. Later on in the manuscript, experiments are conducted on first instar larvae without explanation for the choice of stage. Providing the rationale for using different larval stages would improve clarity.

This is a very interesting point raised by Rev2. Most of our previous work on self-righting (e.g. PicaoOsorio et al. 2015 Science; Picao-Osorio, Baldaia et al. 2017 Genetics; Klann et al. 2021 Journal of Neuroscience) was focused on the first instar larva (L1) because this early stage: (i) represents the simplest form of all larval stages, (ii) allows meaningful comparisons with late embryonic processes guiding the development and physiology of the nervous system, (iii) captures the system in a relatively naïve state, that had limited if any exposure to external stimuli. Although these attributes remain valid for the investigation of the sensory stimuli that trigger self-righting, the implementation of the necessary regional physical measurements and manipulations used in this study (surface contact, opto-axial technique, deep neural network analysis) would be impossible to implement in the early forms of the larva simply due to its reduced size. Due to this, we employed L3s, which due to their larger dimensions enabled the development and use of the sophisticated regional stimulation techniques reported here. Yet, as Rev2 rightly points out, we return to the late embryo and early L1 at the point of conducting gene expression analyses as these are optimised for those early stages. The selection of larval stage according to experiment relies on the fact that all forms of the larva display self-righting (Issa, Picao-Osorio, et al. 2019 Current Biology), that SR does not differ according to larval stage and that the characterisation of the structure of the nervous system across larval stages has shown a large level of similarity and consistent topographically arranged connectivity between identified neurons (Gerhard et al. 2017 eLife).

(2.2) What was the genotype of the larvae used for the initial behavioral characterization (Figure 1)? It is assumed they were wild type or w1118, but this should be stated explicitly. This also raises the question of whether different wild-type strains exhibit this behavior consistently or if there is variability among them. Has this been tested?

Thank you to the reviewer for pointing this out. The genotype for Figure 1 was *w^1118^*; this has now been added to the figure legend and the results section – thank you to Rev2 for pointing this out. Although in this study we did not explicitly compare self-righting (SR) performance in wild type/control genotypes (as we are internally consistent in using *w^1118^*) based on previous data collected in our lab we know that self-righting times are similar and very consistent amongst inbred control lines such as *w^1118^*, *yw*, and *Oregon Red*. Furthermore, we can also add that when comparing SR times between these inbred populations with a highly polymorphic outbred *Drosophila* population (Martins et al. 2013 PLoS Pathogens) we observed that their SR time (i.e. 6.14s ± 1.06) was not significantly different from the inbred lines (p<0.05, U test) (Picao-Osorio, J. 2014 Doctoral Thesis, Chapter 4, p112).

(2.3) Could the observed slight leftward bias in movement angles of the tail (Figure 1I and S1) be related to the experimental setup, for example, the way water is added during the unlocking procedure? It would be helpful to include some speculation on whether the authors believe this preference to be endogenous or potentially a technical artifact.

This is an interesting comment, and we recognise that lateral manipulation biases in self-righting could indeed reflect experimental limitations or biological tendencies. At this point we cannot interpret these results as formal evidence of chirality, given that they may reflect subtle aspects of the micromanipulation of specimens. We are currently developing a motorised platform to conduct self-righting tests, which when fully developed, should help addressing the chirality question.

(2.4) The genotype of the larvae used for Figure 2 experiments is missing.

Thank you for pointing this out. These were again *w^1118^* larvae; this detail has now been added to the figure legend and the main text.

(2.5) The experiment shown in Figure 2E-G reports the proportion of larvae exhibiting self-righting behavior. Is the self-righting speed comparable to that measured using the setup in Figure 1?

Thank you for pointing this out. We have now added average self-righting times to the figure legends of figures 1 and 2. The self-righting times across for the dorsal + ventral contact conditions was notably longer than dorsal-only cases, which were also slightly longer than the “standard” case. This is perhaps to be expected, as the larvae are encountering unusual and ambiguous situations. We suggest the extra time could reflect an additional decision-making step or action flip-flopping process, or simply physical constraints on the movement (for example, not being able to use some parts of the body).

(2.6) Line 496 states: "However, the effect size was smaller than that for the entire multidendritic population, suggesting neurons other than the daIVs are important for self-righting". Although I agree that this is the more parsimonious hypothesis, an alternative interpretation of the observed phenomenon could be that the effect is not due to the involvement of other neuronal populations, but rather to stronger Gal4 expression in daIVs with the general driver compared to the specific one. Have the authors (or someone else) measured or compared the relative strengths of these two drivers?

We agree with this suggestion and to address this concern, we have added as part of our new figure Supp. Fig. S3, a dedicated panel S3C showing fluorescence measurements from ddaC using the 109(2)80-Gal4 and ppk-Gal4 lines. We found no difference in tdTomato fluorescence intensity, suggesting equal expression strength across the two Gal4 drivers. Our new results for axial daIV inhibition are also consistent with this effect size difference, further suggesting that inhibition of all md neurons poses stronger challenges for self-righting compared to the daIV neurons alone.

(2.7) Is there a way to quantify or semi-quantify the expression of the Hox genes shown in Figure 6A? Also, was this experiment performed more than once (are there any technical replicates?), or was the amount of RNA material insufficient to allow replication?

Unfortunately, we only had limited amounts of mRNA extracted from FACS-sorted *109(2)80>GFP* cells to feed our reverse transcriptase reactions and used much of these samples for the experiment reported. After Rev2 suggestion we went back to our freezers, recovered traces of the samples used in the original experiment, and attempted a new amplification; despite this effort, this new experiment was unsuccessful. We feel that the main point deduced from the original experiment is valid in that we obtained amplicons of the expected size for all the Hox transcripts analysed and that for those cases in which we observed biological effects – i.e. Antp and Abd-B – we corroborated protein expression in the 109(2)80 domain using immunohistochemistry. We are currently expanding this project examining the roles of all Hox genes across the entire sensory system and shall report the expression patterns of all Hox genes in each of the subcomponents of the sensory system the future.

(2.8) Since RNAi constructs can sometimes produce off-target effects, it is generally advisable to use more than one RNAi line per gene, targeting different regions. Given that Hox genes have been extensively studied, the RNAis used in Figure 6B are likely already characterized. If this were the case, it would strengthen the data to mention it explicitly and provide references documenting the specificity and knockdown efficiency of the Hox gene RNAis employed. For example, does Antp RNAi expression in the 109(2)80 domain decrease Antp protein levels in multidendritic anterior neurons in immunofluorescence assays?

We used the TRiP RNAi lines, specifically the Valium10 selection available from the Bloomington Stock Centre. Unfortunately, there is not much information on how specific the Hox RNAi lines areor whether their might have off-target effects.

(2.9) In addition to increasing self-righting time, does Antp downregulation also affect head casting behavior or head movement speed? A more detailed behavioral characterization of this genetic manipulation could help clarify how closely it relates to the behavioral phenotypes described in the previous experiments.

This would be interesting line of investigation. As described in a previous comment, this is currently unfeasible for us given some important differences between experiments including larval stage and recording conditions. We have added some speculative comments to the manuscript describing the larval behaviour under Hox RNAi.

(2.10) Does down-regulation of Antp in the daIV domain also increase self-righting time?

Given the new results with axial effects of daIV neurons, we also sought to address this point with a new series of experiments expressing Hox RNAi constructs in the ppk-Gal4 domain. The new data is shown in a new figure (Figure S8) displaying self-righting times for ppk-Gal4-Hox-RNAi. Interestingly, we found no effect of any RNAi expression on self-righting times, suggesting that md types other than daIVs are under Hox regulation that is important for self-righting.

**Recommendations for the authors:**

**Reviewing Editor Comments:**

The reviewers were enthusiastic about the value and quality of this study by Roseby and colleagues. There were two main issues that emerged from the reviews that we're highlighting for the authors to address, should they choose to:

(1) A little more cell-type resolution of the anterior region

The anterior region includes a lot of sensory neurons that may be contributing to the effect. Some sensory neurons (e.g., daIV) have been implicated in righting - are these the ones carrying the anterior signal? Are dorsal sensory neurons promoting righting and ventral ones stalling it?

We are not suggesting a complete sensory-neuron mapping in the anterior region. Instead, we propose the authors conduct a focused check: repeat the axial inhibition with a daIV-specific driver (same photomask assay) to show the A-P effect within the implicated class, and, if possible, replicate one key result with an alternative broad md driver to address Gal4 strength/off-target expression.

As mentioned above (see Rev1 comment) we have indeed carried out a new experiment using an alternative driver to *109(2)80-Gal4* and testing the impact of these manipulations on larval behaviour. The revised version of our MS includes a new figure Supp Fig S3 which shows self-righting times when using the *ppk-Gal4* driver with the opto-axial technique. As with the *109(2)80-Gal4* driver, self-righting was delayed in anterior but not posterior inhibition conditions, suggesting the daIV neurons specifically act in a region-specific manner to trigger postural control behaviour.

Furthermore, in another new figure, Supp Fig S7, we show that head casting behaviour is also increased in the same manner as with the *109(2)80-Gal4* driver. These new panels and figures are cited within the sub-sections entitled “Optogenetic inhibition of anterior but not posterior multidendritic neurons delays self-righting” and “Inhibition of anterior multidendritic neurons is associated with increased head casting during self-righting”, on pages 25 and 28, respectively. We are grateful to R1 for this suggestion, which we consider qualitatively improves the quality of our paper.

(2) The Hox section to strengthen this section, we recommend:

(a) Confirm specificity/efficacy of knockdown (e.g., Antp protein reduction in targeted md neurons and a second RNAi line if available).

This is a reasonable comment. For our experiments, we selected a *UAS-Antp^RNAi^* line (Bloomington #27675) given that this construct has been: (i) utilised in several previous studies as the main and single line to interfere with Anpt expression (e.g. Baek et al. 2013 Development; Paul et al. 2021 Nature Comms) and (ii) shown to display a consistent reduction in Antp protein levels of approximately 50% (see Poliacikova et al. 2024 Science Adv.). Furthermore, previous work comparing #27675 with other *UAS-Antp^RNAi^* lines has demonstrated that all available lines lead to a similar level of reduction in protein expression, although the #27675 line exhibits the most consistent effects (lower variability) (Poliacikova et al. 2024 Science Adv.). Unfortunately, at this point in time, we do not have the capacity to conduct new experiments with other RNAi lines, but consider that the information and arguments mentioned above should be reassuring about our choice of a reasonable and previously validated method to interfere with Antp expression.

(b) Perform one temporal control (GAL80^ts) or a simple rescue, to separate developmental vs acute roles.

This is a good and interesting suggestion, but we consider that the discrimination between developmental and physiological effects falls outside the scope of this study. Indeed, experiments of this kind are currently being conducted in our lab as part of a wider examination of Hox gene roles in the sensory system.

(c) Place the results clearly in the context of prior work (e.g., Parrish 2007), so the mechanism isn't left hanging.

This is an important point, and we have now done this. Many thanks for pointing this out.

**Reviewer #1 (Recommendations for the authors):**

(1.1) A Gal4 line for the pannier dorsal specification gene shows expression in dorsal sensory neurons, as described in Galindo et al., Development, 2023, and could help tease apart dorsal v. ventral contributions.

This is an interesting suggestion. However, we understand that the *pannier* (*pnr*) Gal4 line mentioned in Galindo et al. 2023 is an enhancer trap inserted in the *pnr* locus which drives expression in neural as well as non-neural tissues such as the embryonic dorsal ectoderm (see: Calleja et al. 1996 Development; Stronach et al. 2014 Genetics). Although, as Rev1 rightly indicates, this line also labels dorsal cluster sensory neurons, including ddaC (cIV) and ddaF (cIII) neurons the fact that the line displays expression in non-neural tissues makes its use in behavioural experiments difficult as non-neural effects might affect the behavioural patterns studied. A possible way to instrument the pnrGal4 tool into behavioural analyses might involve the creation of the necessary variants to implement a split-Gal4 approach, but this, we believe, unfortunately falls out of the scope of this study.

(1.2) Potential roles for daII neurons and daI neurons are not examined. Drivers have been described for daII neurons, and there are drivers that will target a majority of proprioceptive md neurons, so these could be examined to complete the analysis started here.

This is another interesting suggestion by Rev1, but we consider that the fine-grain mapping of effects mediated by sensory neuron sub-clases falls outside the scope of this study aimed at mapping sensory regional effects on self-righting. This does not take the merit of the suggestion away, and indeed, experiments of this kind are currently being conducted in our lab as part of a comprehensive examination of Hox gene roles in the sensory system.

(1.3) To account for 109(2)80 off targets, the authors could consider other lines that silence most or all md neurons (clh201-Gal4; 5-40-Gal4; 21-7-Gal4) that could at least have different central offtargets. Some other lines are broad somatosensory system drivers but sensory-specific (pebbledGal4).

This is an interesting comment, and so are the suggestions made. Although to include this kind of verification would be interesting, when carrying out our experiments, we did not observe any central expression at all. Also, to repeat all our experiments in which we use the established and validated 109(2) 80 line using instead these four Gal4 lines, is unfortunately out of scope for us at this point in time. We will nonetheless consider these comments by Rev1 in future extensions of our work.

(1.4) There is a typo on line 481; it should be "other".

We are grateful to R1 for pointing this out. This has now been amended

**Reviewer #2 (Recommendations for the authors):**

(2.1) Lines 91-92 cite references describing self-righting behavior across different animal groups, which is illustrated in Figure 1B. It would be helpful to indicate these references directly in the figure. For example, instead of using dots to denote their presence (which are, in a way, redundant since the behavior is reported in all groups), numbers or letters could be used to refer to the specific papers describing them.

Thank you for this suggestion. We have now replaced the original dots by an abridged citation of a key paper providing evidence in that specific animal group, e.g. Smith, et al. 1997; Rogers et al. 2015

(2.2) In Figure 1A, the diagrams illustrate the two large dorsal tracheae, which nicely indicate the larva's orientation. However, since they are drawn in a very light gray, they can be difficult to distinguish without zooming in. It might improve clarity if the tracheae were made slightly more prominent.

Thank you for this suggestion. We have now implemented this change.

(2.3) In Figure 1E, the dotted line and green bar mark the segment of the recording corresponding to self-righting, which is then quantified in Figure 1G. Was the same procedure applied when analyzing tail speed, or was it limited to head speed? Figure 1F does not show a dotted line or green bar, which is confusing; it would be helpful to clarify the reason for this discrepancy. Also, in Figure 1G, there is an inset showing photos of the movement sequence with the green bar and the caption 'Trimmed to SR sequence,' which implies to me that for tail speed, the 0.75-1 segment of the recording was also used for quantification. I suggest adding the dotted line and green bar to Figure 1F and removing this inset from Figure 1G, as it appears quite small and disrupts the layout of the figure. If it is retained, the figure legend should explicitly refer to the inset.

Thank you for pointing this out. We have amended these figures as suggested.

(2.4) In Figures 1 and 2, the box plots include the individual data points, whereas Figures 3 and S2 do not. For data transparency, it would be important to show the individual measurements here as well. I strongly recommend adding them to the figure, or alternatively providing a clear rationale in the text for not doing so.

Thank you for mentioning this. The reason data points are not shown in Fig 3 or S2 is because the variance extends the scale and compresses the box making it illegible. To make this clear we now explain this in the figure legends.

(2.5) In Figures 4 and 5, the distribution of self-righting times from the optogenetic inhibition experiments is shown using bar graphs rather than box plots, as in the previous figures. This choice obscures the data distribution, since all bars reach down to zero. Replacing the bar graphs in Figures 4 and 5 with box plots would more clearly convey the experimental results.

We thak Rev2 for this comment, which gives us an opportunity to clarify the matter. Distributions of SR times are drawn with bars because we compare means +/- variance in the analysis, and not medians +/- IQR as is done in the other experiments. The choice of visualisation reflects the analysis, which is what is recommended by statisticians. Plus, we also show the individual observations, meaning the distribution can be observed. We hope that it is now clear that we are not obscuring any distributions.

(2.6) Figure 6 would benefit from some reorganization. Panel A is very small and dense with information, making it difficult to interpret without significant zooming. In particular, the FACS graph is nearly impossible to read, as the axes remain unclear even when enlarged. It might be best to either remove this graph and replace it with a cartoon version of FACS-sorted populations, and reorganize the figure to ensure legibility. Additionally, the current layout progresses from the bottom up, which takes time to follow. Comprehension could be improved if the sequence began with the larva dissection placed in the top left area of the figure, where readers typically look first (I appreciate that this is mentioned in the figure legend; however, a different layout might present the information more effectively).

We appreciate the constructive spirit of this comment and have indeed considered Rev2 suggestions including drafting new layouts of this figure. After all this experimentation, we remain of the view that the original presentation is probably the best trade-off between size and clarity, offering more space for the appreciation of confocal imaging and its interpretation.

Minor corrections:

(1) Throughout the text, the word Drosophila appears sometimes in italics and sometimes in regular font; please standardize its formatting for consistency.

Amended

(2) Line 179: the use of three hyphens in the sentence "minimum --- in all cases < 30 s --- to avoid larval desiccation" is unusual; exchanging them for commas or brackets is advised.

Amended

(3) Line 183: in w1118, the numbers are usually in superscript (not subscript), and the w should be italicized.

Amended

(4) In line 783, there is an incorrect space between "is" and the comma in "...repertoire, which is , in...".

Amended

(5) In Figure 2G, the left panel appears partially cut off, which makes the text at the edges difficult to read. It might help to adjust the panel so that all labels are fully visible.

Done

(6) In the current version of the manuscript, Figure 5 is presented before Figure 4, which is confusing.

This has been amended.

(7) Two videos are included in the supplementary material, but I could not find any reference to them in the main text of the manuscript.

This has been amended.
